# Demographics of Patients with Double-headed Pterygium and Surgical Outcomes

**DOI:** 10.4274/tjo.56514

**Published:** 2015-12-05

**Authors:** Fulya Duman, Mustafa Köşker

**Affiliations:** 1 Atatürk State Hospital, Clinic of Ophthalmology, Antalya, Turkey; 2 Ulus State Hospital, Clinic of Ophthalmology, Ankara, Turkey

**Keywords:** Pterygium, double-headed pterygium, conjunctival autograft

## Abstract

**Objectives:**

To analyze demographic and ophthalmologic characteristics of patients with double-headed pterygium in the Mediterranean region of Turkey and to evaluate their surgical outcomes.

**Materials and Methods:**

Records of all patients who underwent surgery for pterygium in Antalya Atatürk State Hospital between November 2012 and March 2014 were retrospectively reviewed. Patients with pterygia on both sides of the cornea (nasal and temporal) were included in the study. Patients with less than six months of follow-up were excluded. Age, occupation and smoking status of patients, recurrence of pterygium and any existing complications in records were evaluated. Fibrovascular proliferation more than 0.5 mm over the cornea was accepted as recurrence.

**Results:**

Eight (5%) of 158 patients who underwent pterygium surgery were diagnosed with double-headed pterygium. Six (75%) of the patients were male and two (25%) were female. Mean age was 42.63 (26-71) years. It was recorded that all patients had worked under the sun for at least 5 hours a day. No intra-operative or post-operative complications were found. Mean follow-up time after surgery was 12 (6-21) months and no recurrence was detected.

**Conclusion:**

Pterygium, especially double-headed pterygium is mostly seen in warm climates and individuals who work outdoors. Dividing the free conjunctival autograft into two and suturing in place of the excised pterygium on both sides of the cornea is a good choice in these patients.

## INTRODUCTION

Pterygium is a common fibrovascular proliferative disease affecting the ocular surface and may cause problems such as irritation and vision problems.^1,2^ According to a meta-analytic study by Liu et al.^3^ including research from twelve countries, the worldwide incidence of pterygium is 10.2%, and the report provides various data regarding the prevalence, risk factors and histopathology of the disease. Although the causes of pterygium are not fully understood, ultraviolet light is believed to be closely related to its development.^4,5^ Pterygium incidence is higher in equatorial areas.^3^ Southern China has the highest incidence of pterygium in the normal population at 33%, compared to the lowest incidence rate in Australia, 2.8%.^6,7^

Many studies have determined the most common risk factors are advanced age, male gender, low education level, high systolic pressure, living in rural areas, dry eye, working outdoors and cigarette use.^3,8,9,10^ The Mediterranean region of Turkey has geographic and climatic high-risk factors for pterygium.

According to Duke-Elder,^11^ pterygium occurs without exception on the nasal side of the conjunctiva, and many studies supporting that assertion were later published. Maloof et al.^12^ attributed this to light coming to the temporal cornea being focused on the nasal cornea. Pterygia on the temporal side are very rare and should be differentiated from squamous cell neoplasms.^13^ Double-headed pterygium, or the development of both nasal and temporal pterygia in the same eye, is rare; in a study by Dolezalova^14^ the incidence was found to be 2.5%.

This study investigates the incidence of double-headed pterygium and patients’ demographic and clinical characteristics in the Mediterranean region of Turkey.

## MATERIALS AND METHODS

One hundred fifty-eight patients recommended for pterygium surgery during routine outpatient clinic examination at the Antalya Atatürk State Hospital between November 2012 and March 2014 were screened, and 8 patients with double-headed pterygium were included in the study. Patients with follow-up periods shorter than 6 months were not included. The age, gender, occupation, cigarette use, time of presentation to the hospital, pterygium grade, time of surgery, visual acuity and other ophthalmologic examination findings of all patients were analyzed retrospectively. Pterygium was graded according to corneal involvement (grade I: passes the limbus; grade II: between the limbus and the pupil; grade III: reaches the pupillary margin; grade IV: crosses the pupillary margin). The study was approved by the Antalya Research and Training Hospital Ethics Committee.

**Figure 1:**
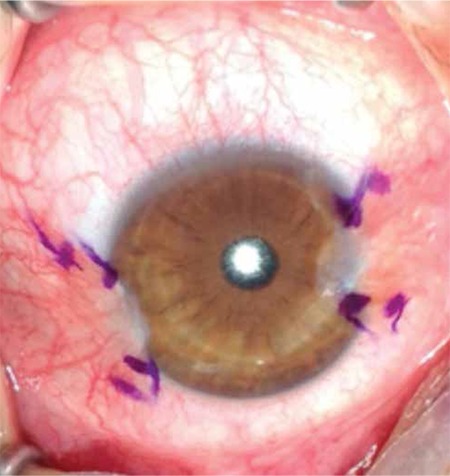
Pterygium of case 2 marked prior to grafting

**Figure 2:**
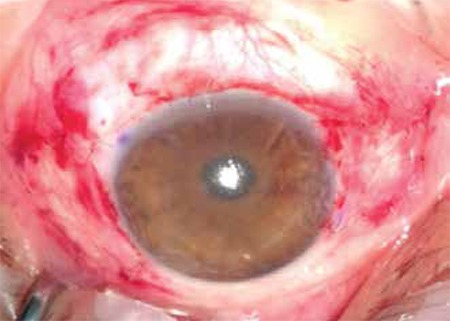
Photograph of case 2 after pterygium surgery with conjunctival autograft

To ensure standardization, patients whose surgeries were conducted by the same surgeon (F.D.) were included. The surgical procedure was routinely performed as follows for all patients: After getting the patient’s medical history and performing routine ophthalmologic examination, the patient was prepared for surgery. For one week prior to the surgery the patient applied topical 0.05% cyclosporin A (Restasis, Allergan Pharmaceutical, Irvine, CA, USA) twice daily. The surgical site was cleaned and 0.5% propacaine hydrochloride (Alcaine, Alcon Laboratories, Inc., Forth Worth, TX, USA) was instilled as topical anesthesia. The nasal and temporal pterygia were marked with a sterile pen and measured, after which local anesthesia (subconjunctival lidocaine hydrochloride + 2% epinephrine) was applied (Figure 1). First, the head of the nasal pterygium was lifted from the corneal surface using toothed forceps and excised. Subconjunctival fibrovascular tissue was dissected with Westcott tenotomy scissors, then hemostasis was achieved by minimally cauterizing the scleral bed using wet field cautery. The limbal and corneal surfaces were scraped smooth with a scalpel. After performing the same procedure on the temporal pterygium, a single piece of conjunctival tissue was taken from the upper bulbar conjunctiva of the same eye and divided into two pieces based on the measurements of the removed pterygia. Agents utilized to inhibit recurrence such as mitomycin C, 5-fluorouracil or triethylene thiophosphoramide were not used in any of the cases.^15,16,17^ The conjunctival grafts were positioned leaving no open scleral tissue starting from the nasal and temporal limbus, with the epithelial side up and the limbal area aligned with the limbus. Both grafts were sutured to the surrounding conjunctiva with a shallow pass through the episclera using 8/0 vicryl (Figure 2). The removed pterygium tissue was send for pathological analysis. Photographs were taken of the eyes before and after surgery for documentation purposes.

All eyes were kept closed for 24 hours after surgery. Treatment with 0.5% loteprednol etabonate+tobramycin (Zylet, Bausch and Lomb, Rochester, NY, USA) and nepafenac (Nevanac, Alcon, Forth Worth, TX) four times daily; topical 0.05% cyclosporin A (Restasis, Allergan Pharmaceutical, Irvine, CA, USA) and bacitracin+neomycin sulfate ophthalmic ointment (Thiocilline, Abdi İbrahim, İstanbul, Turkey) twice daily was continued for 6 weeks postoperatively. Patients were advised to avoid dusty and sunny conditions and to use sunglasses and hats. Patients were asked to return for follow-up at 1 week and at 1, 2, 3 and 6 months. Pterygium recurrence, complications and side effects were analyzed at follow-up. Fibrovascular growth on the cornea greater than 0.5 mm was considered recurrence.

Pre- and postoperative visual acuities were converted to LogMAR before statistical analysis. Paired t-test was used for continuous variables. Rates were compared using Fisher’s exact test. P values under 0.05 were accepted as statistically significant. 

## RESULTS

**Figure 3:**
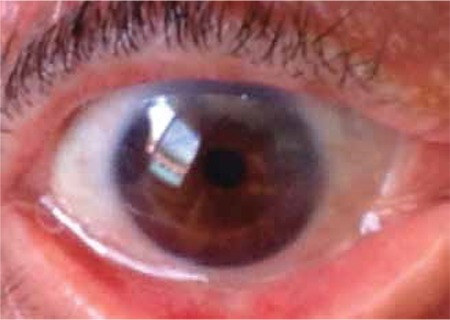
Photograph of case 2 at 3 month follow-up

Of 158 patients who underwent pterygium surgery, 8 patients with pterygium on both sides of the cornea (5% of all the pterygium surgeries) were included in the study. Six of the patients were men and two were women; the mean age was 42.63 years (range, 26-71 years). All patients except one had grade II nasal pterygium and grade I temporal pterygium. The nasal pterygium of case 3 was grade III. No intraoperative or postoperative complications were observed (Figure 3). The mean postoperative follow-up period was 12 months (range, 6-21 months) and there were no recurrences. Histopathologic findings from all excised tissue were consistent with pterygium. All patients reported working at least 5-6 hours per day under the sun during the hottest part of the day, or in the case of a restaurant worker, in front of a fire; homemakers worked in their own gardens or in greenhouses. The rate of cigarette use among the patients was 62.5%. The demographic and clinical characteristics of the patients are presented in Table 1.

Pain, photophobia, tearing and foreign body sensation were not recorded in the early postoperative period, and no statistically significant differences were found in patients’ preoperative and postoperative visual acuity (Table 2).

## DISCUSSION

Pterygium is more common in hot climates. Turkey, especially its Mediterranean region, has the climatic conditions and environmental features that factor in pterygium etiopathogenesis, so pterygium is an important ocular surface disease frequently encountered in our clinic. Despite being a very common ocular pathology, temporal pterygia are quite rare.^13^ The role of pterygium in the development of squamous cell neoplasias is not clear; therefore, excisional biopsy to rule out squamous cell neoplasia should be performed, especially with atypical localization like temporal pterygium. Double-headed pterygium, present on both the temporal and nasal sides of the cornea, is even rarer.^14^ Double-headed pterygium accounted for 5% of all pterygium cases in this study, which is higher than rates reported in other studies. In addition to the regional climate, our patients’ occupations also had a large effect on this rate. All of the patients, including the housewives, reported working at least 5 to 6 hours under the sun during the hottest part of the day (or in front of a fire, in the case of a restaurant worker). Previous studies have also shown higher incidence and increased severity of pterygium in agricultural workers and farmers.^10^ Threlfall and English4 demonstrated a strong association between sun exposure and pterygium. They concluded that sun exposure should be avoided as much as possible and emphasized that all ages should use hats or sunglasses for eye protection.

Although in earlier studies cigarette use has been determined as a risk factor for pterygium, Rong et al.^15^ suggested that cigarette use lowered pterygium risk, especially in chronic cigarette users. In our study the rate of cigarette use was 66.5%. In addition to this factor, advanced age, male gender, low education level, high systolic blood pressure, living in rural areas and dry eye have also been associated with increased pterygium incidence.^3,8,9,10^ In the current study, 75% of the patients were male, the nature of their occupations indicated low education level.

Intraoperative or postoperative complications did not occur in any of the eyes in the study. This is primarily attributed to the decision not to use recurrence inhibiting agents such as mitomycin C, 5-fluorouracil and triethylene thiophosphoramide during surgery. Although using these agents is thought to reduce rates of recurrence, these treatments are known to lead to serious complications such as palpebral hyperpigmentation, scleral and corneal thinning and ulceration, secondary glaucoma, cataract, uveitis, and even globe perforation.^16,17,18,19^

The most important limitation of this study is the small study size; however, as the incidence of double-headed pterygium in the population is extremely low (0.6-2.5%), the number of cases in our study seems adequate.^14,20^ As previous studies have shown that pterygium recurrence typically occurs in 4 to 5 months, this study only included patients with follow-up duration of at least 6 months, but no recurrences were observed.^21,22,23,24^ Of course, future multi-center studies including larger patient numbers and longer follow-up duration will be more enlightening.

In this study we determined the incidence of double-headed pterygium in our region and the demographic and clinical characteristics of these patients. In addition, we observed no recurrence or complications in our patients during the 6-month follow-up period, which indicates that using a bisected autograft taken from the upper bulbar conjunctiva and not using an agent to inhibit recurrence is an appropriate approach in the surgical removal of double-headed pterygium.

## Figures and Tables

**Table 1:**
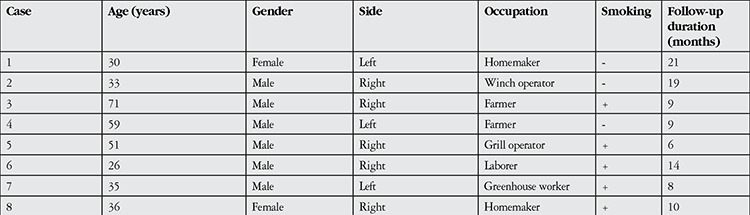
Demographic and clinical characteristics of patients who underwent surgery for double-headed pterygium

**Table 2:**
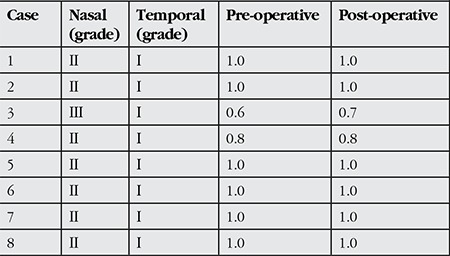
Pterygium grades and pre- and post-operative corrected visual acuity (Snellen) of double-headed pterygium patients
